# Potential Association Between Basal Metabolic Rate and Presbyopia: A Two-Sample Mendelian Randomisation Study

**DOI:** 10.3390/genes17091001

**Published:** 2026-08-25

**Authors:** Young Lee, Je Hyun Seo

**Affiliations:** 1 Veterans Medical Research Institute, Veterans Health Service Medical Centre, Seoul 05368, Republic of Korea; lyou7688@gmail.com; 2 Department of Ophthalmology, Veterans Health Service Medical Centre, Seoul 05368, Republic of Korea

**Keywords:** presbyopia, Mendelian randomisation, basal metabolic rate, ageing, genome-wide association study

## Abstract

**Background/Objectives**: Basal metabolic rate (BMR) is implicated in age-related phenotypes, and presbyopia represents a hallmark of ocular ageing. However, the association between BMR and presbyopia remains underexplored. Therefore, using a two-sample Mendelian randomisation (MR) approach, the present study aimed to evaluate the potential causal relationship between BMR and presbyopia in individuals of European ancestry. **Methods**: Instrumental variables comprised single-nucleotide polymorphisms associated with BMR at genome-wide significance (*p* < 5.0 × 10^−8^), derived from genome-wide association study summary statistics from the UK Biobank. Summary statistics for presbyopia were obtained from the FinnGen project. Causal estimates were primarily assessed using the inverse-variance weighted method and further evaluated using the weighted median method, MR–Egger regression, and the MR–Pleiotropy Residual Sum and Outlier test. **Results**: Genetically predicted higher BMR, expressed per 1-standard-deviation increase on the inverse-rank-normalised scale, was associated with lower odds of presbyopia. The inverse-variance weighted analysis yielded an odds ratio (OR) of 0.79 (95% confidence interval [CI]: 0.67–0.93; *p* = 0.004), with a directionally consistent estimate from the weighted median analysis (OR = 0.76, 95% CI: 0.59–0.99; *p* = 0.045). MR–Egger and SIMEX-corrected MR–Egger analyses yielded estimates in the same inverse direction, although their 95% CIs included the null (OR = 0.73, 95% CI: 0.50–1.05; *p* = 0.093 and OR = 0.71, 95% CI: 0.47–1.06; *p* = 0.092, respectively). The MR-PRESSO global test was nominally significant (*p* = 0.049), although no individual outlier was identified. In additional MVMR analyses incorporating BMI or standing height, the direct effect of BMR was not statistically significant in either model. **Conclusions**: These findings suggest a potential inverse association between higher genetically predicted BMR and the odds of presbyopia. However, MVMR analyses did not support a direct effect of BMR independent of BMI or height, warranting caution in interpreting the observed association as specific to metabolic rate. Further studies are required to validate these results and clarify the underlying biological mechanisms.

## 1. Introduction

Presbyopia is an age-related decline in the ability of the eye to focus (accommodative function) that affects nearly all individuals in mid to late adulthood and represents a major cause of visual discomfort [1,2]. The condition has been traditionally attributed to progressive biomechanical and structural changes in the crystalline lens and ciliary body, which reduce lens elasticity and diminish accommodative amplitude [3]. Despite this well-established framework, the age at onset and rate of progression vary considerably among individuals, suggesting that factors beyond local ocular ageing may contribute to its development [4].

Accumulating evidence indicates that age-related ocular disorders may reflect broader systemic ageing processes [5]. Alterations in energy metabolism, mitochondrial function, and oxidative stress contribute to age-related dysfunction across multiple tissues, including neural and muscular systems. In this context, basal metabolic rate (BMR), which is the minimum energy expenditure required to sustain vital physiological processes at rest, serves as an integrative indicator of systemic metabolic activity and a proxy for biological ageing [6]. Consistent with this framework, genetic and epidemiologic studies have reported associations between BMR and several age-related phenotypes, including frailty, cognitive decline, sarcopenia, and mortality [7,8]. Although BMR generally declines with advancing age, with an accelerated rate of decline in older individuals, a paradoxically blunted age-related decrease in BMR is associated with increased mortality [9]. A recent study also reported a positive association between BMR and cognitive function [10]. Collectively, these findings highlight the need for a more in-depth evaluation of the implications of BMR.

Given that presbyopia is a well-established age-related condition, investigating its association with BMR may provide valuable insights into the metabolic determinants of ocular ageing. However, to date, the influence of systemic metabolic rate on age-related accommodative decline has not been systematically investigated. A study on the epidemiology of presbyopia [11] revealed that factors influencing its prevalence include sex (female; odds ratio [OR] = 1.19, 95% confidence interval [CI]: 1.02–1.45), exposure to pollutants (fine particulate matter with an aerodynamic diameter ≤ 2.5 µm and ozone), socioeconomic conditions, and nutrition. Moreover, inadequate nutritional status, including deficiencies in essential amino acids, has been recognised as a potential risk factor [12], with prior research suggesting that antioxidant nutrients may help prevent age-related changes in the crystalline lens [13]. However, evidence on these metabolic factors in presbyopia has largely relied on observational designs, which are susceptible to residual confounding and reverse causation. Therefore, it remains unclear whether variation in BMR contributes causally to accommodative dysfunction or merely reflects concurrent age-related changes. Moreover, the causal nature of the association between BMR and presbyopia cannot be reliably inferred from conventional epidemiologic approaches.

Mendelian randomisation (MR) leverages genetic variants associated with an exposure of interest as instrumental variables (IVs) to strengthen causal inference [14,15]. Given that genetic variants are randomly allocated at conception, MR analyses are less susceptible to confounding and reverse causation than conventional observational studies. In recent years, several studies employing MR techniques for risk factor analysis in ophthalmological research have been published [16,17]. Therefore, in the present study, we aimed to clarify whether systemic energy metabolism contributes to the pathogenesis of age-related accommodative decline. To this end, we used a two-sample MR framework based on large-scale genome-wide association study (GWAS) data to evaluate the potential causal association between BMR and presbyopia.

## 2. Materials and Methods

### 2.1. Study Design

The study protocol was approved by the Institutional Review Board of the Veterans Health Service Medical Centre (IRB No. 2026-01-034); the requirement for informed consent was waived, owing to the retrospective study design. All procedures adhered to the tenets of the Declaration of Helsinki.

### 2.2. Data Sources

**Figure 1:**
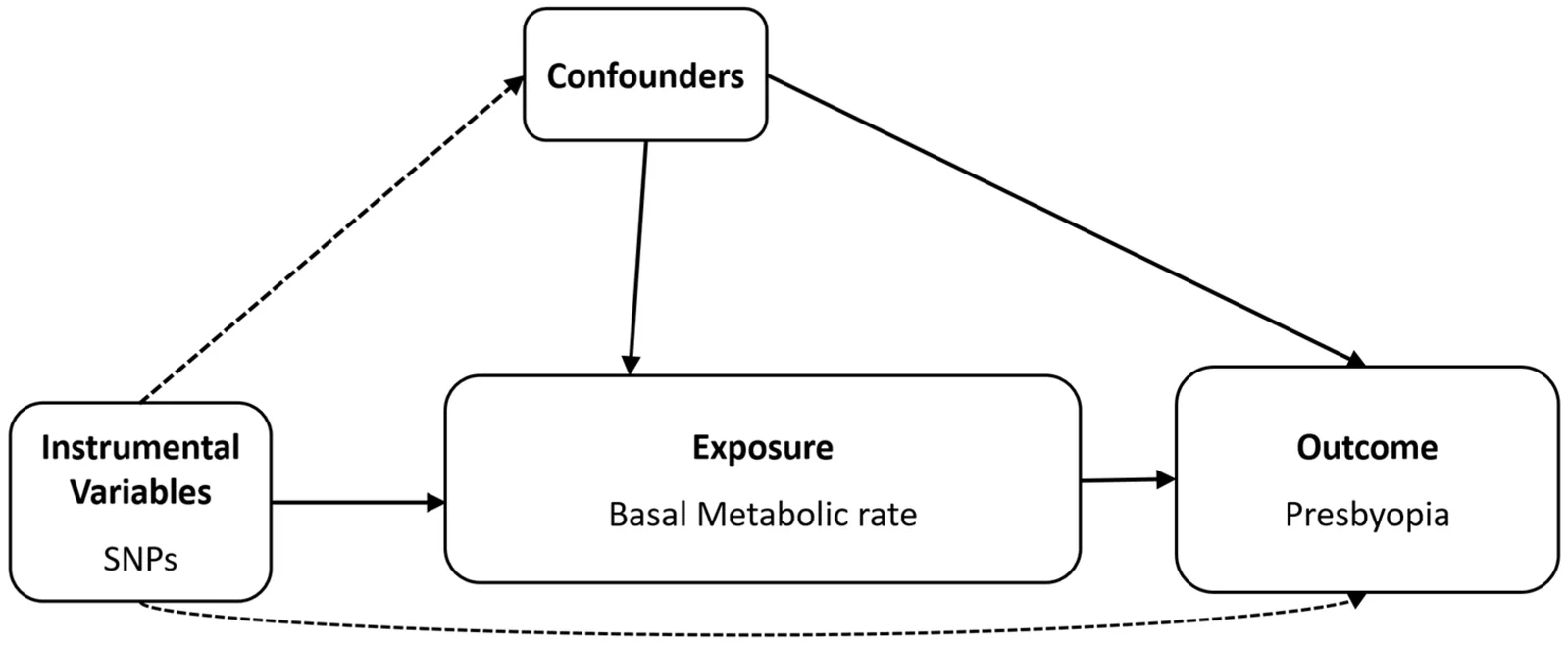
Diagram of two-sample Mendelian randomisation analysis. Solid arrows indicate associations that should be present, whereas dashed arrows indicate associations that should be absent according to the assumptions of Mendelian randomization. SNP, single-nucleotide polymorphism.

Figure 1 presents a schematic overview of the analytical study design for investigating the potential causal effect of BMR on the risk of presbyopia. Exposure data were obtained from the both-sexes European-ancestry GWAS summary statistics in the Pan-UK Biobank (*n* = 413,178; https://pan.ukbb.broadinstitute.org/downloads, accessed on 17 February 2026, Table 1). BMR corresponded to UK Biobank field 23105, “Basal metabolic rate” a continuous phenotype derived from bioelectrical-impedance-based body-composition assessment and originally recorded in kilojoules. In the Pan-UK Biobank GWAS, BMR was inverse-rank normal transformed within the European-ancestry group before association testing. For the additional MVMR analyses, GWAS summary statistics for body mass index (BMI) and standing height were also obtained from the corresponding both-sexes European-ancestry Pan-UK Biobank datasets. BMI corresponded to UK Biobank field 21001 (*n* = 419,163), and standing height corresponded to UK Biobank field 50 (*n* = 419,596). These anthropometric traits were used to evaluate whether the association between genetically predicted BMR and presbyopia was independent of body size. Outcome data were obtained from GWAS summary statistics for the H7_PRESBYOPIA endpoint in FinnGen Release 12 (*n* = 479,081, including 2305 cases and 476,776 controls; https://finngen.gitbook.io/documentation/data-download; accessed on 22 February 2026, Table 1). Presbyopia cases were identified from national health registry records based on ICD-10 code H52.4 and the corresponding ICD-9 code 3674 and ICD-8 code 37002 recorded in the hospital discharge or cause-of-death registries. Thus, the outcome represented registry-recorded presbyopia rather than self-reported reading glasses use or direct measurement of accommodative function. Individuals meeting the broader H7_OCUMUSCLE endpoint, which includes disorders of ocular muscles, binocular movement, accommodation, and refraction, were excluded from the control group. Other ocular conditions, including myopia, hyperopia, and astigmatism, were not specifically excluded from the presbyopia case group. Because only GWAS summary statistics were available, individual-level age, sex, and ocular-comorbidity distributions could not be examined or used to apply additional exclusions.

### 2.3. Selection of Genetic Instrumental Variables (IVs)

Single-nucleotide polymorphisms (SNPs) associated with BMR at the GWAS threshold (*p* < 5.0 × 10^−8^) were selected as candidate IVs. Palindromic SNPs (A/T or G/C) were excluded to avoid ambiguity in strand alignment. To ensure independence among the IVs, SNPs were clumped using a stringent linkage disequilibrium threshold of *r*^2^ < 0.001 within a 10,000 kb window. These parameters were intentionally selected to minimise residual correlation among the instruments and corresponded to the default stringent clumping settings implemented in the TwoSampleMR framework. The 1000 Genomes Project Phase III European reference panel was used for linkage disequilibrium estimation during clumping. The exposure and outcome datasets were subsequently harmonised using the harmonise_data function in the TwoSampleMR package to align effect alleles and strand orientation and to ensure that genetic associations corresponded to the same effect allele across datasets. To confirm the instruments were sufficiently strong, we examined the F-statistic; exceeding the commonly used cutoff of 10 was regarded as indicating a low risk of weak-instrument bias [18].

### 2.4. MR Analysis

The MR analysis was grounded in three core assumptions for IVs: (1) the genetic instruments must be strongly associated with the exposure; (2) they must not be associated with confounders of the exposure–outcome relationship; and (3) The underlying assumption is that no independent pathway links the instruments to the outcome other than through the exposure, thereby signifying an absence of directional horizontal pleiotropy.

As the primary analytic strategy, we employed the inverse-variance weighted (IVW) approach with a multiplicative random-effects specification to estimate causal relationships [19,20,21]. To assess the robustness of the findings, complementary approaches, including the weighted median estimator [22], MR–Egger regression with and without simulation extrapolation (SIMEX) correction [23,24], and the MR–Pleiotropy Residual Sum and Outlier (MR–PRESSO) test, were also applied [25].

The IVW method provides reliable estimates when all genetic variants satisfy the IV assumptions [26], whereas violations of these assumptions may introduce bias [22]. Consistency of causal estimates under the weighted median approach is preserved even when up to half of the instrumental variables fail to meet validity criteria [22]. The MR–Egger technique allows estimation of causal effects in the presence of pleiotropy by permitting a non-zero intercept, which reflects the average horizontal pleiotropic effect [23]. The *I*^2^*_GX_* statistic was used to assess potential regression dilution in MR–Egger analysis under the no measurement error assumption. An *I*^2^*_GX_* value greater than 90% indicates that substantial regression-dilution bias is unlikely. Although the observed *I*^2^*_GX_* value exceeded 90%, MR–Egger with SIMEX correction was additionally performed as a conservative sensitivity analysis [24]. The MR-PRESSO global test was used to assess evidence of overall horizontal pleiotropy, and the outlier test was used to identify individual variants contributing disproportionately to pleiotropy. When one or more outliers were identified, an outlier-corrected estimate was calculated after their removal, and the distortion test was applied to determine whether the causal estimates differed significantly before versus after outlier correction [25]. When no statistically significant outlier was identified, the outlier-corrected estimate and distortion test were not applicable. Accordingly, the results were interpreted based on the most appropriate MR method [27].

Heterogeneity among IVs was assessed using Cochran’s Q statistic for IVW and Rücker’s Q′ statistic for MR–Egger [19,28]. Horizontal pleiotropy was evaluated using the MR–Egger intercept test and the MR–PRESSO global test. A *p*-value < 0.05 for Cochran’s Q, Rücker’s Q′, or the MR–PRESSO global test was considered indicative of potential heterogeneity or pleiotropy. To further assess whether the association between genetically predicted BMR and presbyopia was independent of major anthropometric traits, additional multivariable Mendelian randomisation (MVMR) analyses were performed. Separate two-exposure models jointly evaluated BMR with BMI or height. Conditional F-statistics were calculated to assess instrument strength for each exposure conditional on the other exposure in the model, with values >10 considered indicative of adequate conditional instrument strength [18]. Horizontal pleiotropy and residual heterogeneity were assessed using the Q_A_ statistic, a modification of Cochran’s Q statistic for MVMR [18]. When the Q_A_ statistic indicated significant heterogeneity, direct effects were estimated using the modified Q-statistic minimisation approach [18], with 95% CIs obtained using jackknife resampling.

All MR effect estimates are reported as ORs with 95% CIs and represent the change in the odds of presbyopia per 1-standard-deviation increase in genetically predicted BMR on the inverse-rank-normalised scale. Because BMR was analysed on an inverse-rank-normalised scale in the Pan-UK Biobank GWAS, the estimates cannot be directly translated into a specific increase in BMR measured in kilojoules. A two-sided *p*-value < 0.05 was considered statistically significant. Statistical power was evaluated using the mRnd web-based power calculator (https://shiny.cnsgenomics.com/mRnd/; accessed on 7 August 2026) [29], incorporating the proportion of variance in BMR explained by the genetic instruments, the outcome sample size and case proportion, and the effect estimate from the primary IVW analysis. All MR analyses were performed using the TwoSampleMR, MVMR, MendelianRandomization, and simex packages in R (version 3.6.3; R Foundation for Statistical Computing, Vienna, Austria).

## 3. Results

### 3.1. Genetic Instrumental Variables in MR

We identified 594 independent genetic variants associated with BMR at genome-wide significance (*p* < 5.0 × 10^−8^) and used them as IVs (Table 2). The mean F-statistic was 80.15, and all individual F-statistics exceeded the conventional threshold of 10, indicating a low risk of weak instrument bias (Table 2 and Table S1). Detailed information on the selected IVs is provided in Table S1.

We assessed heterogeneity using Cochran’s Q test for the IVW model and Rücker’s Q′ test for the MR–Egger model. Cochran’s Q test suggested borderline heterogeneity among the instruments (*p* = 0.0503; Table 2), whereas Rücker’s Q′ test indicated significant heterogeneity (*p* = 0.048; Table 2).

The *I*^2^*_GX_* statistic was 92.03%, indicating that substantial regression dilution in the MR–Egger estimate was unlikely. Therefore, the MR–Egger analysis with SIMEX correction was interpreted as an additional conservative sensitivity analysis. Regarding horizontal pleiotropy, the MR–PRESSO global test suggested possible overall horizontal pleiotropy (*p* = 0.049). However, the MR–PRESSO outlier test did not identify any statistically significant outlying variants; therefore, no outlier-corrected estimate or distortion-test result was available.

The MR–Egger intercept did not indicate evidence of directional horizontal pleiotropy, both before (*p* = 0.621) and after SIMEX correction (*p* = 0.563). Given the evidence of heterogeneity and potential pleiotropy, we used the IVW method with multiplicative random effects as the primary MR analysis [27]. Nevertheless, residual heterogeneity or horizontal pleiotropy could not be excluded, and the IVW estimate was interpreted together with the sensitivity analyses.

### 3.2. MR for the Causal Effects of BMR on Presbyopia

Genetically predicted higher BMR was associated with lower odds of presbyopia. In the multiplicative random-effects IVW analysis, the OR for presbyopia was 0.79 (95% CI: 0.67–0.93; *p* = 0.004). The estimated statistical power to detect an association of this magnitude was 89%. The weighted median analysis yielded a directionally consistent estimate (OR = 0.76, 95% CI: 0.59–0.99; *p* = 0.045). The MR–Egger analysis revealed a similar, not statistically significant, direction of effect (OR = 0.73, 95% CI: 0.50–1.05; *p* = 0.093), with consistent results after SIMEX correction (OR = 0.71, 95% CI: 0.47–1.06; *p* = 0.092).

**Figure 2:**
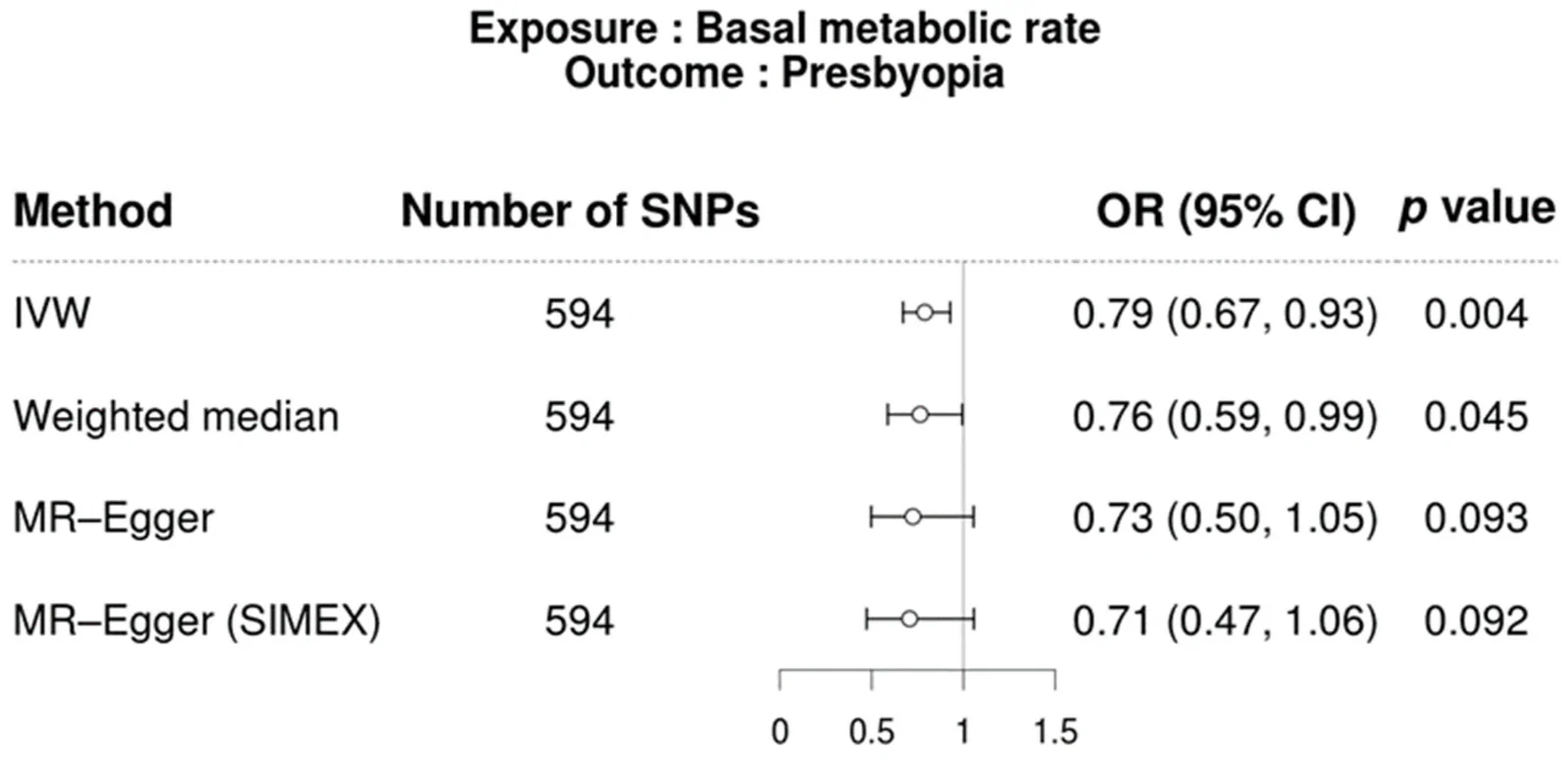
Forest plot of Mendelian randomisation estimates for the association between basal metabolic rate and presbyopia. CI, confidence interval; IVW, inverse-variance weighted; MR, Mendelian randomisation; OR, odds ratio; PRESSO, pleiotropy residual sum and outlier; SIMEX, simulation extrapolation; SNP, single-nucleotide polymorphism.

**Figure 3:**
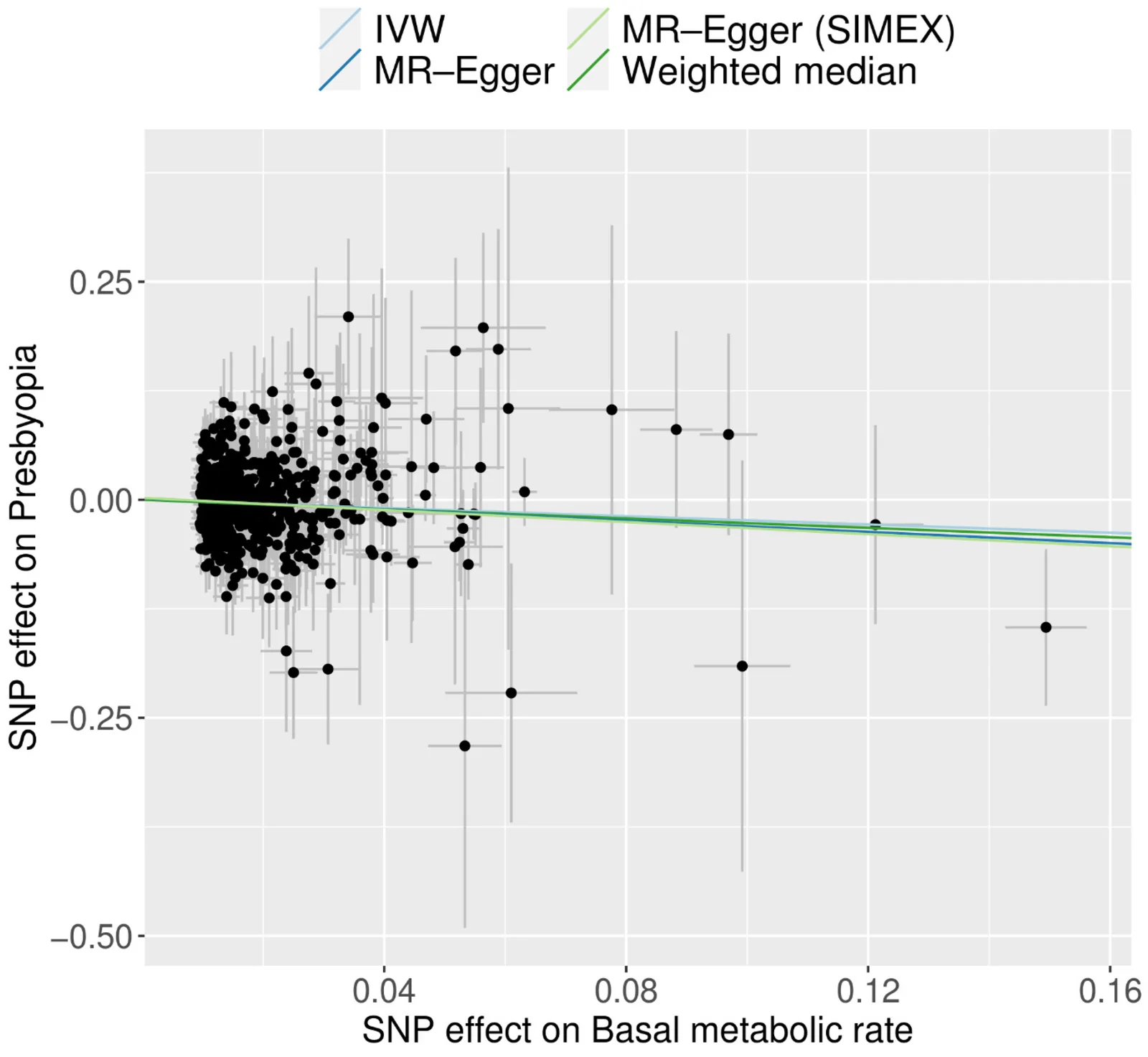
Scatter plot of SNP-specific associations and Mendelian randomisation estimates for basal metabolic rate and presbyopia. Light blue, dark blue, light green, and dark green regression lines represent the IVW, MR–Egger, MR–Egger (SIMEX), and weighted median estimates, respectively. The slope of each regression line represents the MR estimate obtained using the corresponding method. Each point represents a SNP, with the genetic association with the exposure shown on the x-axis and that with the outcome shown on the y-axis. IVW, inverse-variance weighted; MR, Mendelian randomisation; PRESSO, pleiotropy residual sum and outlier; SIMEX, simulation extrapolation; SNP, single-nucleotide polymorphism.

As presented in Figure 2, causal estimates across different MR methods were generally consistent in direction. Although the MR–Egger and SIMEX-corrected MR–Egger estimates were not statistically significant, their point estimates remained in the same inverse direction as those from the IVW and weighted median analyses. The wider confidence intervals of the MR–Egger estimates may reflect the relatively lower statistical precision of MR–Egger regression compared with other MR approaches [23]. The SNP-specific associations between BMR and presbyopia are illustrated in the scatter plot (Figure 3).

In the additional MVMR analyses, the conditional F-statistics exceeded 10 for both exposures in the BMR–BMI and BMR–height models, indicating adequate conditional instrument strength (Supplementary Table S2). The Q_A_ statistic indicated significant residual heterogeneity in both models (Supplementary Table S2). Accordingly, direct effects were estimated using the modified Q-statistic minimisation approach, with 95% CIs obtained using jackknife resampling. In the BMR–BMI model, the direct-effect estimates were OR = 0.94 (95% CI: 0.66–1.34) for BMR and OR = 1.07 (95% CI: 0.78–1.46) for BMI. In the BMR–height model, the corresponding estimates were OR = 1.28 (95% CI: 0.86–1.93) for BMR and OR = 0.84 (95% CI: 0.66–1.07) for height. All 95% CIs included the null value of 1.0 (Table 3).

## 4. Discussion

BMR reflects the systemic energy expenditure required to maintain essential physiological processes, including mitochondrial activity, cellular maintenance, and redox homeostasis. Growing evidence suggests that metabolic regulation plays a critical role in the ageing [30]. Additionally, recent studies have reported associations between body fat distribution and biological ageing [31], and another study has suggested a possible link with cardiovascular ageing [32]. Furthermore, another study has reported that body mass, in association with hypertension, may serve as a predictive factor for neural ageing [33]. Metabolomic studies of the ageing eye have demonstrated substantial alterations in mitochondrial metabolism, glucose utilisation, and methylation pathways across ocular tissues, including the retina, optic nerve, and lens, collectively indicating that metabolic dysregulation is a key feature of ocular ageing [30]. In light of this perspective, we aimed to investigate the relationship between accommodative muscle aging (presbyopia) and anthropometric/body composition features. The present study provides a potential association between BMR and presbyopia in European populations, based on two-sample MR analyses. Using 594 independent SNPs robustly associated with BMR, genetically predicted elevated BMR was associated with a decreased risk of presbyopia in individuals of European ancestry, with significant associations observed using both the IVW and weighted median methods.

One hypothesis linking BMR to presbyopia involves oxidative stress and mitochondrial function, given the eye’s high metabolic activity and continuous exposure to light-induced oxidative stimuli [34]. In the crystalline lens, age-related depletion of antioxidant defenses, particularly glutathione, has been proposed to promote protein aggregation and crystallin cross-linking, potentially contributing to lens stiffening and impaired accommodation [35,36]. On this basis, individuals with genetically elevated BMR might, in principle, exhibit more efficient metabolic turnover and cellular repair, which could plausibly attenuate oxidative damage to the lens. We emphasize, however, that this pathway remains speculative and cannot be directly evaluated using our data. Our MR analysis demonstrates only a modest inverse genetic association between BMR and presbyopia risk, without established biological specificity, and this finding should not be interpreted as evidence of a protective mechanism. Notably, as discussed above, the genetic instruments for BMR are also associated with body composition traits, raising the equally plausible possibility that the observed association reflects a non-ocular, systemic pathway (e.g., related to body fat or lean mass) rather than a lens-specific oxidative-stress mechanism. Furthermore, BMR elevation driven by pathological states such as hyperthyroidism reflects distinct physiology from the genetic determinants used as instruments here, and the two should not be conflated. Given these uncertainties, the mechanistic explanation offered above should be regarded as hypothesis-generating rather than an established biological pathway, and dedicated mechanistic studies are needed to clarify whether, and how, systemic metabolic activity influences lens ageing.

An important consideration in interpreting the present findings is the close relationship between BMR and body size. The UK Biobank BMR phenotype shares substantial genetic architecture with anthropometric traits. To examine whether the univariable BMR association was independent of body size, we additionally performed MVMR analyses incorporating BMI or standing height. Although conditional instrument strength was adequate, the direct effect of BMR was not statistically significant in either model. These findings do not support a BMR-specific effect independent of BMI or height and suggest that the univariable association may, at least in part, reflect genetic pathways shared between BMR and anthropometric traits. Given the strong genetic correlations among these phenotypes, however, the MVMR results should not be interpreted as demonstrating that BMI or height mediates or fully explains the observed univariable association.

The primary strength of this study lies in the use of a large cohort dataset, enabling a robust MR analysis and supporting a potential causal association between BMR and presbyopia. Leveraging summary statistics from large-scale GWAS consortia minimised confounding and reverse causation, common limitations of conventional observational studies. The IVs used in our study were strong, as reflected by high F-statistics, and multiple MR approaches were applied to address pleiotropy and heterogeneity. Nevertheless, certain limitations warrant consideration. First, the analysis was restricted to individuals of European ancestry, limiting generalisability to other populations. In addition, although both GWAS datasets were of European ancestry, the exposure and outcome data were derived from different European populations. Population-genetic differences between the UK Biobank and FinnGen, including differences in allele frequencies and LD structure, may have affected the transferability of the genetic instruments. Second, although MR minimises confounding, pleiotropy cannot be fully excluded, particularly given the complex genetic architecture of metabolic traits. Third, BMR is a composite phenotype influenced by body composition, endocrine factors, and genetic determinants; therefore, the specific biological mediators underlying the observed association remain unclear. Finally, presbyopia was defined using registry-recorded diagnostic codes rather than direct measurements of accommodative function. Therefore, individuals with presbyopia who did not seek medical care or receive a registry-recorded diagnosis may not have been captured as cases, potentially resulting in outcome misclassification. In addition, other ocular conditions, including myopia, hyperopia, and astigmatism, were not specifically excluded from the presbyopia case group and may have contributed to phenotype heterogeneity. Because only GWAS summary statistics were available, detailed age, sex, and ocular-comorbidity distributions could not be compared between cases and controls, and additional individual-level exclusion or subgroup analyses were not possible.

## 5. Conclusions

The present findings align with emerging evidence that metabolic phenotypes influence age-related traits. Although BMR generally declines with age, inter-individual variation in genetically determined metabolic rate may reflect biological ageing trajectories rather than chronological ageing. The observed inverse association raises the possibility that genetically elevated BMR may be related to the functional ageing of accommodative structures. The MR design reduces the confounding and reverse causation that typically limit observational studies, thereby strengthening inference of a potential causal relationship. These results support a role for body mass features in ocular ageing processes. Future studies integrating longitudinal clinical data, detailed ocular biometric measurements, and mechanistic investigations are warranted to clarify the biological pathways linking metabolic rate and accommodative decline.

## Figures and Tables

**Table 1:** Summary statistics of the data source.

Traits	Data Source	No. of Participants	Population	No. of Variants	Reference
BMR	UKB	413,178	European	23,079,601	Pan-UK Biobank (https://pan.ukbb.broadinstitute.org/downloads/index.html, accessed 17 February 2026)
BMI	UKB	419,163	European	23,111,080	
Height	UKB	419,596	European	23,113,399	
Presbyopia	FinnGen	479,081 (2305 cases + 476,776 controls)	European	21,326,809	https://finngen.gitbook.io/documentation/data-download; accessed 22 February 2026

BMI, body mass index; BMR, basal metabolic rate; UKB, UK Biobank.

**Table 2:** Heterogeneity and horizontal pleiotropy of instrumental variables.

Exposure				Heterogeneity			Horizontal Pleiotropy						
									MR-Egger			MR-Egger (SIMEX)	
	N	F	*I*^2^*_GX_* (%)	*p* *	*p* ^#^		*p* ^†^		Intercept, β (SE)	*p*		Intercept, β (SE)	*p*
BMR	594	80.15	92.03	0.05	0.048		0.049		0.0017 (0.0035)	0.621		0.0022 (0.0037)	0.563

*I*^2^*_GX_*: statistic assessing potential regression dilution in MR–Egger analysis under the no measurement error assumption; *: Cochran’s Q test from IVW; ^#^: Rücker’s Q’ test from MR–Egger; ^†^: MR–PRESSO global test; β, beta coefficient; F, mean F-statistic; IVW, inverse-variance weighted; MR, Mendelian randomisation; N, number of instruments; PRESSO, pleiotropy sum of residuals and outlier; SE, standard error; SIMEX, simulation extrapolation; BMR, basal metabolic rate.

**Table 3:** Multivariable Mendelian randomisation analyses of basal metabolic rate and anthropometric traits in relation to presbyopia.

Model	Exposure	OR (95% CI)
Model 1	BMR	0.94 (0.66, 1.34)
Model 1	BMI	1.07 (0.78, 1.46)
Model 2	BMR	1.28 (0.86, 1.93)
Model 2	Height	0.84 (0.66, 1.07)

## Data Availability

The datasets used and/or analysed in the current study are publicly available from the Pan-UK Biobank (https://pan.ukbb.broadinstitute.org/downloads/index.html; accessed 17 February 2026) and FinnGen (https://finngen.gitbook.io/documentation/data-download; accessed 22 February 2026).

## Supplementary Materials

The following supporting information can be downloaded at https://www.mdpi.com/article/10.3390/genes17091001/s1, Table S1: List of single-nucleotide polymorphisms used as instrumental variables in the univariable Mendelian randomisation analysis; Table S2: Instrument strength and heterogeneity diagnostics for the multivariable Mendelian randomisation analyses.

## Abbreviations

The following abbreviations are used in this manuscript:

BMR	Basal Metabolic Rate
OR	Odds Ratio
CI	Confidence Interval
MR	Mendelian Randomisation
GWAS	Genome-Wide Association Study
SNPs	Single-Nucleotide Polymorphisms
IVW	Inverse-Variance Weighted
SIMEX	Simulation Extrapolation
MR–PRESSO	Mendelian Randomisation–Pleiotropy Residual Sum and Outlier
